# Bilateral Thalamic Infarction Secondary to Thrombosis of Artery of Percheron

**DOI:** 10.7759/cureus.13707

**Published:** 2021-03-04

**Authors:** Abdullah Shams, Syed Ahmed Hussaini, Fateen Ata, Mohamed Abdelhady, Mohammed Danjuma

**Affiliations:** 1 Internal Medicine, Hamad Medical Corporation, Doha, QAT; 2 Internal Medicine, CMH Lahore Medical and Dental College, Lahore, PAK; 3 Psychiatry, Hamad Medical Corporation, Doha, QAT; 4 Neuroradiology Section, Neuroscience Institute, Hamad Medical Corporation, Doha, QAT

**Keywords:** stroke, artery of percheron, bilateral thalamic infarct

## Abstract

The artery of Percheron (AOP) is a rare anatomical variation emerging from the posterior circulation and supplies both thalami in the brain. As per the literature, the AOP infarction constitutes less than 2% of all stroke cases. AOP infarctions are usually caused by a combination of risk factors and a predisposing vascular territory. The areas most affected by AOP are the paramedian thalami with or without the involvement of the midbrain. AOP can be challenging as it is infrequent and mostly can be missed on the initial scans. We present a 58-year-old previously healthy male known to have hypertension with poor follow-up who presented with dysarthria and facial weakness, which he felt after waking up from sleep. After the initial physical examination and investigations, a preliminary diagnosis of stroke was made. As the patient was worked up for the stroke, his symptoms improved, and he was back to his baseline function within 48 hrs of presentation. What came to our surprise was that the stroke workup, including the initial CT scan with an angiogram, blood works (Hba1c and lipid panel), echocardiogram of the heart (ECHO), and Holter monitor was all unremarkable until an MRI head was done, which showed bilateral thalamic acute-sub acute infarct. This shows that AOP can be easily missed as it may not appear on the initial scans and workup and needs an adequate radiological study for diagnosis. Although some cases of AOP infarction are reported in the literature, the presentation with transient mild symptoms makes our case an interesting one.

## Introduction

The thalamus’ blood supply is complex and comprises small end arteries emerging the P1 and P2 segment of the posterior cerebral artery (PCA) and the posterior communicating artery. The thalamic vasculature can be divided into four territories: anterior, paramedian, inferolateral, and posterior. Artery of Percheron (AOP) is a single perforating vessel that is an anatomical variant of paramedian thalamic arterial supply arising from the p1 segment of either PCA and ends up supplying bilateral thalami [1].

The acute artery of Percheron infarcts comprise 0.1 to 2% of all ischemic strokes. However, of all thalamic stroke, occlusion of the artery of Percheron is attributed to around 4-35% of cases. Early detection of AOP can be missed due to its rare occurrence and can be negative on early imaging, including computed tomography or magnetic resonance [2]. Thus, encountering bilateral thalamic infarction in clinical scenarios, AOP should be considered to avoid confusion with a neurological phenomenon like infections and tumors.

## Case presentation

A 58-year-old gentleman, a non-smoker known to have hypertension, presented to the emergency department with new-onset slurring of speech that he noticed at noon after waking up from sleep. The patient had no other past medical condition and reported this as the first episode. According to the patient, he slept well and woke up with the symptoms of slurring of speech and involuntary spilling of water during an attempt to drink, which made him visit the hospital. He denied any headache, vision problems, fever, weakness in upper or lower limbs, loss of consciousness, sphincter incontinence, and sensory symptoms. He had no family history of stroke and denied any drug abuse.

**Figure 1:**
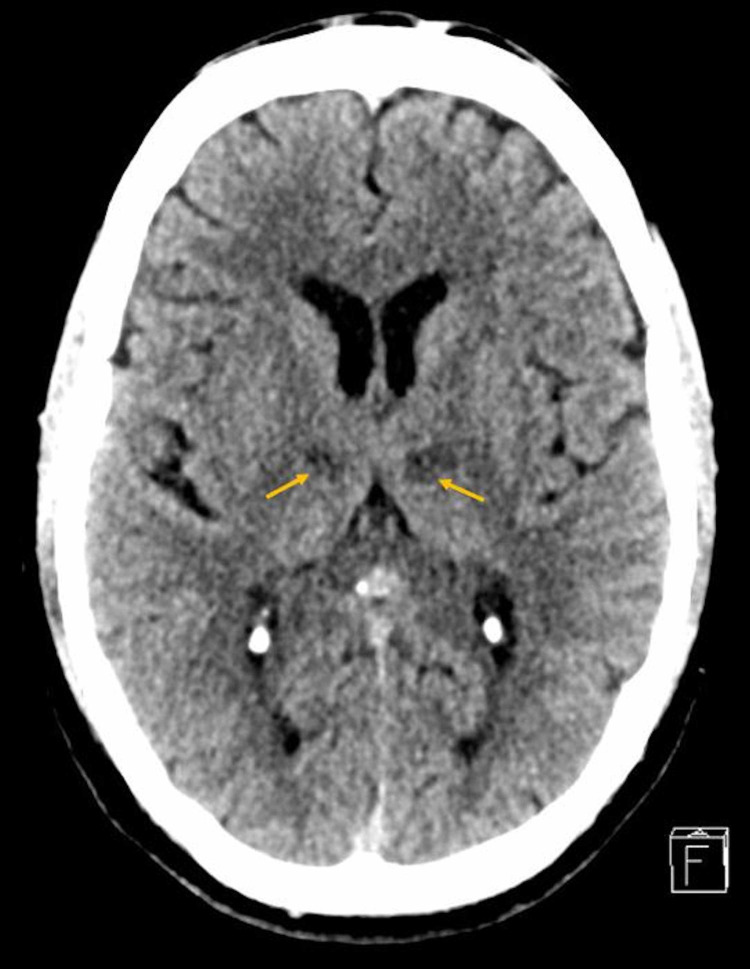
Brain plain CT axial image.

On initial inspection, the patient was afebrile with a heart rate of 75 per minute, blood pressure of 155/85 mm of Hg, and normal oxygen saturation at room air. Physical examination was remarkable for dysarthria, and facial weakness due to the inability to close the mouth or fully blow cheeks or sensory deficit noted in both upper and lower limbs. His reflexes were intact with a normal gait, and no cerebellar signs were present. The rest of the systemic examination was unremarkable as well. Based on these findings, an initial impression of stroke was made, and the patient was urgently sent for computerized tomography (CT) scan and angiogram of the head, which was later reported unremarkable (Figure 1). The patient was started on aspirin and statin, and was shifted to the medical floor to complete the stroke workup.

**Figure 2:**
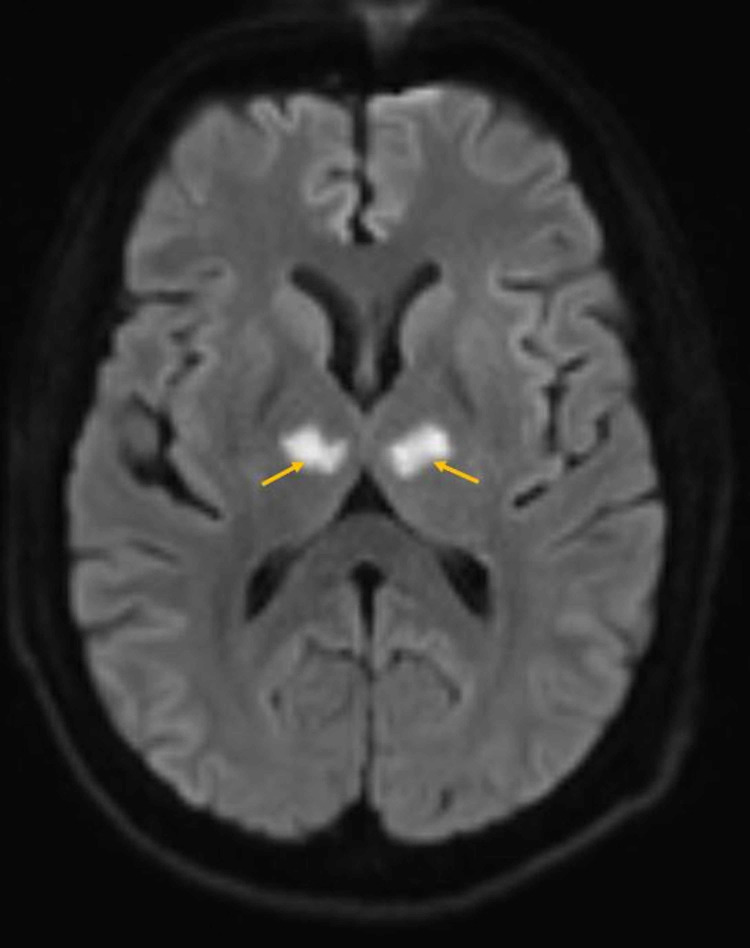
Brain MR diffusion weighted (DWI) axial image. Bilateral anterior thalamic high signal intensity is seen.

**Figure 3:**
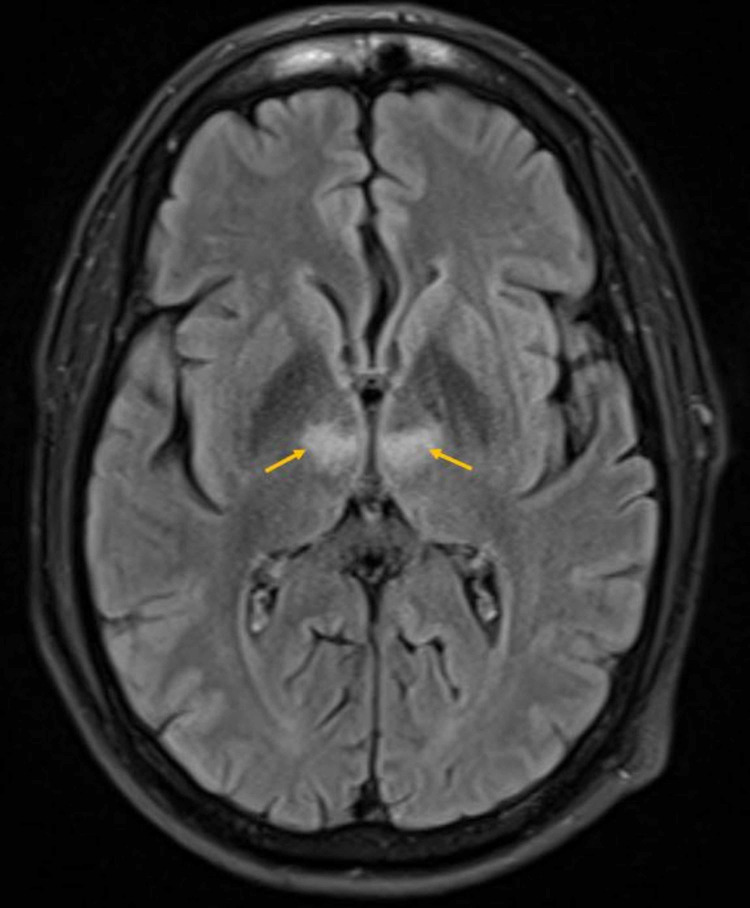
Brain MR fluid-attenuated inversion recovery (FLAIR) axial image. Bilateral anterior thalamic high signal intensity corresponding to the areas of diffusion restriction.

**Figure 4:**
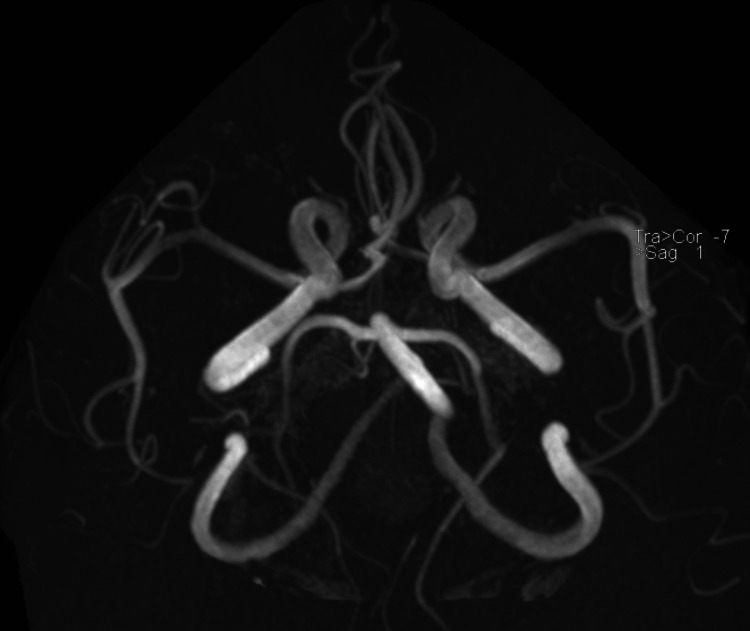
3D time of flight intracranial MRA demonstrating patent major intracranial arteries. The occluded artery of Percheron is not visualized due to the small size and thrombosis. MRA: Magnetic resonance angiography

Throughout the next 48 hours, patient’s symptoms improved entirely, and he was back to his baseline. The stroke workup, including blood work for the lipid panel and HBa1c along with echocardiogram (echo) and Holter monitor, was unremarkable. So far, the diagnosis was going with transient ischemic attack (TIA) until an MRI was done, which displayed bilateral anterior thalamic areas of diffusion restriction (Figure 2) demonstrating corresponding high fluid-attenuated inversion recovery/T2 weighted image (FLAIR/T2WI) signal intensity (Figure 3) features consistent with acute infarcts along artery of Percheron territory. Major intracranial arteries and internal cerebral veins were patent (Figure 4). As the patient was asymptomatic, the findings of bilateral thalamic infarction, too due to the vascular anomaly (AOP), were unusual, ruling out infection or tumor as the cause. After stroke team review, the patient was continued on aspirin and statin along with optimization of antihypertensive management and was discharged with follow-up as an outpatient.

## Discussion

Gerard Percheron first described the artery of Percheron as being one of the four anatomical variants of arterial supply to the paramedian thalami in which one dominant thalamic perforating artery arises from P1 and bifurcates to produce both paramedian thalami and, in some cases, the rostral mesencephalon [3-7]. Artery of Percheron infarct, being an unusual occurrence, has been previously reported specifically for the rarity of the artery of Percheron variant within the population and, therefore, the radiological challenges [8, 9]. Physicians remain in a diagnostic dilemma while treating patients presenting with not-so-clear manifestations of posterior circulation stroke. Occlusion of the artery of Percheron (AOP) leads to a characteristic pattern of bilateral paramedian thalamic infarcts with or without mesencephalic infarctions [9-11].

The spectrum of clinical features evident in AOP infarcts span over seven patterns:

1. Mental status changes, including stupor, somnolence, and coma

2. Aphasia/slurred speech/dysarthria

3. Behavior and memory impairment incorporate confusion, agitation, apathy, disinhibition, hyperphagia, amnesia, and pseudobulbar affect

4. Ocular movement abnormalities which include horizontal, vertical gaze paresis with or without pupillary involvement

5. Cerebellar signs and symptoms like ataxia and dysmetria

6. Motor deficits (often defined as paresis or paralysis of any type affecting the face, upper limbs, or lower limbs)

7. Other nonspecific clinical presentations such as hypersomnia, seizures, and hyperthermia [12].

AOP occlusions present a neurodiagnostic challenge. Several cases are reported on AOP infarction where initial CT was deemed normal [3, 6, 11, 13, 14]. As a result, a correct diagnosis of an AOP occlusion is currently a diagnostic dilemma as the quality of care in managing an acute ischemic stroke depends on timing, anatomical location of lesion, and contraindications to use thrombolytics. Recombinant tissue plasminogen activator (tPA) administered within 4.5 hours of onset combined with mechanical thrombectomy within 6 hours is argued to be the well-liked treatment of proximal cerebral arterial occlusion [15]. The American Heart Association has similar guidelines for stroke occlusion of the proximal middle cerebral or internal arterial blood vessel. Emergent AOP stroke cases should initially be treated with intravenous (IV) heparin and tPA if not contraindicated, followed by subsequent long-term anticoagulation [12]. Those involving the midbrain are treated with IV heparin [16]. Another case reports that IV heparin administration with the activated period maintained between 300 and 350 seconds with a follow-up MRI of the brain showed minimal residual stroke with IV heparin administration [17].

Finally, the treatment of AOP infarction should be focused on the pathophysiology of underlying disease. Long-term oral anticoagulant and antiplatelet therapy with Aspirin and Plavix could be debated and are generally prescribed by clinicians counting on the underlying disease process like cardioembolic events require long-term oral anticoagulants, whereas cryptogenic causes require antiplatelet therapy [12].

## Conclusions

Bilateral infarct being rare, can present with series of symptoms that vary from case to case. AOP should be kept in mind while managing patients with bilateral thalamic infarct having unusual symptoms as modifiable risk factors, if treated, can lead to better outcomes in patient care.
